# Self-Sampling of Oropharyngeal Swabs Among Healthcare Workers for Molecular Detection of Respiratory Viruses: A Valuable Approach for Epidemiological Studies and Surveillance Programs

**DOI:** 10.3389/fpubh.2020.511669

**Published:** 2020-11-23

**Authors:** Cristina Galli, Laura Pellegrinelli, Gabriele Del Castillo, Giovanni Forni, Cecilia Eugenia Gandolfi, Marco Mosillo, Anna Pietronigro, Navpreet Tiwana, Silvana Castaldi, Elena Pariani

**Affiliations:** ^1^Department of Biomedical Sciences for Health, University of Milan, Milan, Italy; ^2^Fondazione Istituti di Ricovero e Cura a Carattere Scientifico (IRCCS) Ca' Granda Ospedale Maggiore Policlinico, Milan, Italy; ^3^Interuniversity Research Center on Influenza and Other Transmissible Infections (CIRI-IT), University of Genoa, Genoa, Italy

**Keywords:** self-sampling, oropharyngeal swabs, respiratory viruses, molecular analysis, epidemiological studies, surveillance

## Abstract

This study aimed at assessing the validity of self-collected (self-sampled) oropharyngeal (OP) swabs among healthcare workers compared to those collected by trained sentinel general practitioners (GP-sampled) from individuals with influenza-like illness (ILI), to be implemented in epidemiological studies and/or surveillance programs of viral pathogens involved in community respiratory infections. In our study, OP swabs were collected from adults (>18 years) with ILI during the 2018–2019 influenza season. Two groups of samples were considered: group 1−131 self-sampled OP swabs collected by healthcare workers after being trained on the sampling procedure; group 2−131 GP-sampled OP swabs collected from outpatients by sentinel GPs operating within the Italian Influenza Surveillance Network. To assess swabbing quality, following RNA extraction, each sample was tested for the presence of the human ribonuclease P gene (RNP) by in-house real-time reverse transcriptase–polymerase chain reaction (RT-PCR). Samples with a cycle threshold (Ct) <35 were considered adequate for further virological analysis. Influenza viruses (IVs), respiratory syncytial virus (RSV), and rhinovirus (RV) genomes were detected by in-house real-time RT-PCR. All samples were positive to RNP detection with Ct <35. The mean Ct value was similar in the two groups (group 1 vs. group 2: 25.93 ± 2.22 vs. 25.46 ± 2.40; *p* = 0.10). IVs, RSV, and RV positivity rates were 26.7 vs. 52.7% (*p* < 0.01), 7.6 vs. 9.9% (*p* = 0.52), and 21.4 vs. 19.9% (*p* = 0.76), respectively. Self-sampled OP swabs resulted as valid as GP-sampled OP swabs for molecular detection of respiratory viruses. Self-swabbing can thus be a worthwhile strategy for sample collection to implement molecular surveillance of respiratory pathogens and carry out epidemiological studies, easily reaching a larger population size.

## Introduction

An adequate specimen collection is essential to identify viral pathogens involved in respiratory infections. A gold-standard sample for viral detection in upper respiratory tract infections is the oropharyngeal (OP) swab, which is usually collected in healthcare structures, such as hospitals, clinics, and practices (1). However, going to see the doctor to collect a swab could be perceived as a waste of time by the patient, particularly if disease symptoms are mild, and the identification of the etiological agent does not improve treatment or prognosis. Collecting respiratory secretions at home could overcome this negative feeling and may increase patient compliance to epidemiological studies and surveillance programs of respiratory pathogens, especially useful for estimating the circulation of those pathogens causing mild diseases, such as influenza-like illness (ILI). In Europe, influenza surveillance relies mainly on sentinel general practitioners (GPs) in charge of recording the number of ILI cases per week and collecting respiratory specimens from their patients for laboratory tests (2). Self-sampling of OP swabs could be a successful, time-, and cost-effective approach for the detection of viruses involved in community respiratory infections and could be a valuable sampling strategy to rapidly assess viral community transmission and maximize containment measures such as patient isolation or quarantine during a respiratory outbreak.

This study aimed at assessing the validity of self-sampled OP swabs compared to those collected by trained sentinel GPs from individuals with ILI in terms of swabbing quality (evaluated by testing an endogenous human gene) and efficiency of molecular detection of respiratory viruses.

## Materials and Methods

### Study Samples and Population

OP swabs were collected from adults (≥18 years) with ILI during the 2018–2019 influenza season (from week 46/2018 to week 17/2019). According to the European Center for Disease Prevention and Control case definition, an ILI case is defined as a sudden onset of symptoms, with at least one systemic (fever or feverishness, malaise, headache, and myalgia) and one respiratory (cough, sore throat, and shortness of breath) symptom (3). Sampling had to be performed within 7 days from symptom onset. OP swabs were collected by means of Sigma Virocult® kit (Medical Wire—MWE, United Kingdom) that consisted in a prepackaged sterile kit containing a small vial with 1 mL transport medium, stable at room temperature, and a dry swab composed by an open-celled foam bud and a stick with a specific breakpoint (4). Once collected, OP swabs were stored at +4°C and sent to the reference laboratory for the Lombardy region (Department of Biomedical Sciences for Health, University of Milan) of the Italian Influenza Surveillance Network (InfluNet) within 72 h.

Two groups of samples were considered and compared:

- Group 1: 131 OP swabs that were self-collected (self-sampled OP swabs) by healthcare workers (including doctors, nurses, technicians, ancillary workers, in-training students, etc.) at a university and research hospital in Milan (Fondazione IRCCS Ca' Granda, Ospedale Maggiore Policlinico) in the framework of a cohort study aimed at estimating the 2018–2019 influenza vaccine effectiveness. Each participant was provided with the swab, along with an explanatory brochure describing the steps of swab collection. If ILI symptoms were developed during the course of the influenza season, each participant was instructed to contact the study staff via phone call: if an ILI was confirmed by the abovementioned definition, the participant had to self-collect the OP swab in accordance with the sampling procedure illustrated in the explanatory brochure provided at the time of enrollment, and following the instructions provided telephonically by the study staff member.

- Group 2: 131 OP swabs that were collected by sentinel GPs (GP-sampled OP swabs) operating within InfluNet in the Lombardy region. These trained physicians collected OP swabs from ILI outpatients seeking medical attention at their practices (5).

OP swabs were collected from as many ILI cases (*N* = 262); the median age of ILI cases in the two groups was similar [group 1: median age, 38.8 years; interquartile range (IQR), 26.5 years; group 2: median age, 43.4 years; IQR, 17.9 years; *p* = 0.94].

### Laboratory Tests

For molecular analyses, RNA was extracted from 200 μL of each swab by a commercial kit (Invisorb® Spin Virus RNA Mini kit, Stratec Biomedical AG, Germany) following the manufacturer's instructions. Each sample was tested for the presence of the endogenous human ribonuclease P gene (RNP) to assess the swabbing quality and the extraction performance. The RNP detection was performed by an in-house real-time one-step reverse transcriptase–polymerase chain reaction (RT-PCR) assay using 5 μL of RNA to be added in 15 μL of reaction mixture (Luna Universal Probe One-Step RT-qPCR kit; New England Biolabs Inc., United States) including a specific set of primers (final concentration: 0.8 μM each) and probe (final concentration: 0.2 μM) (6). The thermal profile was 55°C for 10 min, 95°C for 1 min, 45 cycles at 95°C for 10 s, and 55°C for 30 s. RNP has to be positive for each swab indicating that human cellular RNA/DNA is present in the sample, and the extraction process was successful. The RNP cycle threshold (Ct) value of each tested sample was normalized against a positive control. Only samples with a Ct <35 were considered adequate for further molecular virological analyses.

Study specimens were then tested for the detection of influenza viruses (IVs) types A and B, respiratory syncytial virus (RSV) and rhinovirus (RV) by means of specific in-house one-step real-time RT-PCR assays (7, 8). For all assays, 5 μL of RNA was added to 15 μL of reaction mixture (Luna Universal Probe One-Step RT-qPCR kit; New England Biolabs Inc., United States). The final concentrations of the oligonucleotides were 0.8 μM of IVs primers and 0.2 μM of IVs probes, 0.5 μM of RSV primer forward, 0.25 μM of RSV primer reverse and 0.05 of RSV probe, and 1 μM of RV primers and 0.1 μM of RV probe. The thermal profile was 55°C for 10 min, 95°C for 1 min, and 45 cycles at 95°C for 10 s followed by an annealing/extension phase of 55°C for 30 s for IVs, and 55°C for 1 min for RSV and RV. A sample was considered positive to a viral target when its Ct value was <40. Real-time assays were performed by using a StepOnePlus real-time PCR system (Thermo Fisher Scientific, United States).

### Statistical Analysis

Mean values [and standard deviation (SD)] by group were calculated and compared by means of the unpaired *t*-test. The frequencies of samples positive to a specific target were compared by χ^2^ test. A two-tailed *p* < 0.05 was considered significant.

## Results

All study swabs (*N* = 262) were collected within 7 days from ILI symptoms onset, with a mean time of collection of 2.2 ± 1.5 days for self-sampled OP swabs and 1.8 ± 1.3 days for GP-sampled OP swabs (*p* = 0.02); 95.4% (125/131) of self-sampled OP swabs and 98.5% (129/131) of GP-sampled OP swabs were collected on or before day 5 (mean time of collection, 2.0 ± 1.2 days vs. 1.7 ± 1.1 days; *p* = 0.05).

All OP swabs were positive for the detection of RNP, indicating a good swabbing technique quality and a successful extraction process. All self-sampled and GP-sampled OP swabs had an RNP Ct value <35 cycles, showing that all study specimens resulted adequate for virus detection analysis. The mean RNP Ct value was 25.93 (SD, 2.22; range, 19.48–33.13) for self-sampled OP swabs and 25.46 (SD, 2.40; range, 18.65–30.10) for GP-sampled OP swabs, with no statistically significant difference between the two groups (*p* = 0.10).

The IV positivity rate was 26.7% (35/131) for self-sampled OP swabs and 52.7% (69/131) for GP-sampled OP swabs (*p* < 0.01). All IV-positive swabs were collected within 7 days regardless of the group considered (mean time of collection, 2.4 ± 1.9 days for self-sampled OP swabs vs. 1.8 ± 1.3 days for GP-sampled OP swabs; *p* = 0.15).

The frequency of RSV-positive samples was similar between the two groups: 7.6% (10/131) in self-sampled OP swabs and 9.9% (13/131) in GP-sampled OP swabs (*p* = 0.52). All RSV-positive swabs were collected within 4 days of symptom onset (mean time of collection, 2.5 ± 1.2 days for self-sampled OP swabs vs. 1.7 ± 1.2 days for GP-sampled OP swabs; *p* = 0.18). RV positivity rate was similar between the two groups: 21.4% (28/131) in self-sampled OP swabs and 19.9% (26/131) in GP-sampled OP swabs (*p* = 0.76). The RV-positive swabs were collected within 7 days (mean time: 2.1 ± 1.5 days) for self-sampled OP swabs and within 6 days (mean time, 2.0 ± 1.7 days) for GP-sampled OP swabs (*p* = 0.80).

## Discussion

Surveillance systems of respiratory infections (such as influenza surveillance) usually rely on sentinel physicians who monitor the epidemiological trend of a specific illness and collect respiratory samples from their outpatients for virological analyses (2). In this study, self-swabbing was evaluated as an alternative to trained physician sampling as it may be an effective, time-, and cost-efficient approach for epidemiological studies and surveillance programs.

We compared the self-sampled OP swabs to those collected by trained sentinel GPs and demonstrated that self-swabbing is as valid as the OP sampling by trained physicians to be used in molecular assays to investigate respiratory viruses involved in ILI. We assessed the RNP detection to check the quality of each sample. The positivity for RNP in self-sampled OP swabs as well as in the GP-sampled OP swabs indicates the presence of human cells in all samples, thus revealing that all swabs were taken with enough thoroughness to contain exudate fragments from the back walls of the throat and highlighting the good quality of both sampling approaches. As observed in other studies (9, 10), no difference was observed in this human housekeeping gene Ct values between the self-sampled and the not self-sampled respiratory swabs (*p* = 0.93), suggesting that the two swabbing strategies are equivalent in obtaining adequate samples for molecular virological analyses (9, 10). Moreover, previous studies on acute respiratory infections have demonstrated the feasibility and acceptability of the self-swab sampling approach (11–13) and have verified the quality and efficiency of viral detection of self-collected respiratory swabs, with excellent agreement in viral detection (10, 14). In fact, in these latter studies, no significant difference was observed in the rate of respiratory samples testing positive for a panel of respiratory pathogens obtained from self-sampling vs. clinician-sampling schemes (10–14). Our results confirmed that self-swabbing is a non-inferior sampling strategy compared to trained physician swab collection in terms of viral detection rates, as the frequencies of positive samples to viral target were similar among the two groups. Self-swabbing can thus be used more routinely to supplement or replace trained sentinel GP sampling to assess community transmission of a known organism.

Although the self-sampled OP swabs were collected later compared to the GP-sampled OP swabs, all swabs were collected within 7 days, and the mean time of collection for almost all specimens was similar (~2 days from symptom onset) among the two study groups, suggesting that self-swabbing can be used as much as the trained physician sampling without compromising the timeliness of sample collection in the acute virological phase. Our study also provided the opportunity to evaluate the best collection time of OP swabs from symptom onset in order to investigate respiratory viruses involved in upper respiratory tract infections. While IVs and RV positive swabs were detected up to 7 days after symptoms onset, RSV was identified within 4 days only, probably as a consequence of the different viral shedding (15–17). As the time of sample collection can affect virus detection, for a more sensitive identification of viral targets in respiratory samples, a swabbing period of 4 days instead of 7 days should be preferred. However, further analyses including the detection of a larger panel of respiratory viruses are needed to better clarify this issue.

Particularly, when a surveillance program on new targets is in the pipeline, an *ad hoc* evaluation of the sampling time should be carefully considered. This study highlighted the potential of self-swabbing in the public health setting as a worthwhile strategy of sample collection for scientific research and surveillance programs. Self-swabbing can be a valuable tool for epidemiological studies, monitoring the circulation of viral infectious agents, implementing molecular surveillance of respiratory viruses, or evaluating vaccine effectiveness. This technique allows more easily reaching an appropriate number of enrolled subjects to achieve adequate statistical power.

Furthermore, avoiding a doctor's appointment for swab collection could increase the willingness of individuals to participate in epidemiological studies and surveillance programs of respiratory pathogens, especially to investigate the etiology of mild diseases. Moreover, it can be an opportunity to study respiratory infections in low-income communities (18).

A previous study aimed at investigating the comparative accuracy of paired OP, and nasopharyngeal swabs for molecular diagnosis of a panel of respiratory pathogens has shown that neither specimen was consistently more effective than the other and that the relative performance of specimen type may vary by the investigated virus (19). Self-sampling of OP swabs, or other respiratory specimens, may be a sampling strategy with great potential and impact during pandemics such as the ongoing (at the time of writing) COVID-19 (coronavirus disease 2019); in fact, self-sampling may aid in the rapid assessment of SARS-CoV-2 (severe acute respiratory syndrome coronavirus 2) community transmission among healthcare workers and may maximize containment measures. This could provide pivotal insight for staff management, as well as for establishing and tracking the source of infection and determining infection prevention measures (20, 21).

Self-swabbing can be a practicable sampling strategy, also thanks to technological innovations. Respiratory swabs currently on the market are simple enough to be used independently by an adult patient, following few simple instructions. These swabs usually incorporate a specific liquid transport medium, which is stable at room temperature and guarantees proper conditions for the collection, transport, maintenance, and long-term frozen storage of viruses. Moreover, the use of biomolecular techniques, for the detection of respiratory viruses, which are more sensitive than culture-based assays, makes self-swabbing an efficient sampling strategy to investigate community respiratory infections. In fact, virus detection by cell cultures requires careful storage of the sample, in terms of both time and temperature, in order to keep the pathogen alive. Furthermore, only cultivable viruses can be investigated. Conversely, molecular assays are robust virological investigation techniques that offer the opportunity to potentially detect all viral pathogens, even in small samples or following suboptimal storage conditions.

A limitation of this study was the enrollment of a selected study population, the healthcare workers, as participants for self-sample testing. This population was recruited within a university and research hospital, meaning most of the subjects were probably skilled at swab collection. Further studies considering a larger sample size and, particularly, involving the general population could be considered to strengthen our results.

In conclusion, our results support that self-sampling is a valid method of enhancing community-based epidemiological studies and surveillance programs for molecular detection of respiratory viruses in upper respiratory tract illnesses.

## Data Availability Statement

The datasets generated for this study are available on request to the corresponding author.

## Ethics Statement

The studies involving human participants were reviewed and approved by Comitato Etico Milano Area 2 (n. 1941, 09/10/2018) - Fondazione IRCCS Cà Granda, Ospedale Maggiore Policlinico di Milano. The patients/participants provided their written informed consent to participate in this study.

## Author Contributions

CG, LP, and EP gave substantial contributions to the conception and design of the work. GD, GF, CG, MM, AP, and NT contributed to acquisition, analysis, or interpretation of data for the work. CG and LP performed laboratory tests, contributed to data analysis and interpretation, and drafted the work. SC and EP revised the manuscript critically for important intellectual content. All authors provided approval for publication of the content and agreed to be accountable for all aspects of the work in ensuring that questions related to the accuracy or integrity of any part of the work are appropriately investigated and resolved. All authors contributed to the article and approved the submitted version.

## Conflict of Interest

The authors declare that the research was conducted in the absence of any commercial or financial relationships that could be construed as a potential conflict of interest.

## References

[1] World Health Organization (WHO) Global Influenza Surveillance Network. Manual for the Laboratory Diagnosis and Virological Surveillance of Influenza. (2020). Available online at: http://whqlibdoc.who.int/publications/2011/9789241548090_eng.pdf (accessed November 8, 2019).

[2] European Centre for Disease Prevention and Control (ECDC) European Influenza Surveillance Network (EISN). (2020). Available online at: https://ecdc.europa.eu/en/about-us/partnerships-and-networks/disease-and-laboratory-networks/eisn (accessed November 8, 2019).

[3] European Centre for Disease Prevention and Control (ECDC) Influenza-Like Illness Case Definition. (2020). Available online at: https://ecdc.europa.eu/en/home (accessed November 8, 2019).

[4] Medical Wire Sigma Virocult^®^ *MW951S*. (2020). Available online at: https://www.mwe.co.uk/microbiology-lab-supplies/culture-swabs-liquid/sigma-virocult-mini-mw951s/ (accessed November 8, 2019).

[5] Istituto Superiore di Sanità (ISS) InfluNet: Rete Italiana Sorveglianza Influenza. (2020). Available online at: https://old.iss.it/site/RMI/influnet/Default.aspx (accessed November 8, 2019).

[6] Centers for Disease Control and Prevention (CDC) National Center for Immunization and Respiratory Diseases (NCIRD). Virology, Surveillance, and Diagnosis Branch. (2020). Available online at: https://www.cdc.gov/ncird/flu.html (accessed November 8, 2019).

[7] Lu X Holloway B Dare RK Kuypers J Yagi S Williams JV. Real-time reverse transcription-PCR assay for comprehensive detection of human rhinoviruses. J Clin Microbiol. (2008) 46:533–9. 10.1128/JCM.01739-07 18057136 PMC2238069

[8] Fry AM Chittaganpitch M Baggett HC Peret TC Dare RK Sawatwong P. The burden of hospitalized lower respiratory tract infection due to respiratory syncytial virus in rural Thailand. PLoS ONE. (2010) 5:e15098. 10.1371/journal.pone.0015098 21152047 PMC2994907

[9] Haussig JM Targosz A Engelhart S Herzhoff M Prahm K Buda S. Feasibility study for the use of self-collected nasal swabs to identify pathogens among participants of a population-based surveillance system for acute respiratory infections (GrippeWeb-Plus)-Germany, 2016. Influenza Other Respir Viruses. (2019) 13:319–30. 10.1111/irv.12644 30925029 PMC6586186

[10] Akmatov MK Gatzemeier A Schughart K Pessler F. Equivalence of self- and staff-collected nasal swabs for the detection of viral respiratory pathogens. PLoS ONE. (2012) 7:e48508. 10.1371/journal.pone.0048508 23155387 PMC3498275

[11] Cooper DL Smith GE Chinemana F Joseph C Loveridge P Sebastionpillai P. Linking syndromic surveillance with virological self-sampling. Epidemiol Infect. (2008) 136:222–4. 10.1017/S0950268807008412 17394678 PMC2870802

[12] Plymoth A Rotzen-Ostlund M Zweygberg-Wirgart B Sundin CG Ploner A Nyren O. Self-sampling for analysis of respiratory viruses in a large-scale epidemiological study in Sweden. Euro Surveill. (2015) 20:21063. 10.2807/1560-7917.ES2015.20.11.21063 25811646

[13] Jackson ML Nguyen M Kirlin B Madziwa L. Self-Collected nasal swabs for respiratory virus surveillance. Open Forum Infect Dis. (2015) 2:ofv152. 10.1093/ofid/ofv152 26613095 PMC4653956

[14] Elliot AJ Bermingham A Charlett A Lackenby A Ellis J Sadler C. Self-sampling for community respiratory illness: a new tool for national virological surveillance. Euro Surveill. (2015) 20:21058. 10.2807/1560-7917.ES2015.20.10.21058 25788252

[15] Hall CB Long CE Schnabel KC. Respiratory syncytial virus infections in previously healthy working adults. Clin Infect Dis. (2001) 33:792–6. 10.1086/322657 11512084

[16] Peltola V Waris M Kainulainen L Kero J Ruuskanen O. Virus shedding after human rhinovirus infection in children, adults and patients with hypogammaglobulinaemia. Clin Microbiol Infect. (2013) 19:E322–7. 10.1111/1469-0691.12193 23490188

[17] Suess T Remschmidt C Schink SB Schweiger B Heider A Milde J. Comparison of shedding characteristics of seasonal influenza virus (sub)types and influenza A(H1N1)pdm09; Germany, 2007-2011. PLoS ONE. (2012) 7:e51653. 10.1371/journal.pone.0051653 23240050 PMC3519848

[18] Vargas CY Wang L Castellanos de Belliard Y Morban M Diaz H Larson EL. Pilot study of participant-collected nasal swabs for acute respiratory infections in a low-income, urban population. Clin Epidemiol. (2016) 8:1–5. 10.2147/CLEP.S95847 26793005 PMC4708198

[19] Kim C Ahmed JA Eidex RB Nyoka R Waiboci LW Erdman D. Comparison of nasopharyngeal and oropharyngeal swabs for the diagnosis of eight respiratory viruses by real-time reverse transcription-PCR assays. PLoS ONE. (2011) 6:e21610. 10.1371/journal.pone.0021610 21738731 PMC3128075

[20] Reusken CB Buiting A Bleeker-Rovers C Diederen B Hooiveld M Friesema I. Rapid assessment of regional SARS-CoV-2 community transmission through a convenience sample of healthcare workers, the Netherlands, March 2020. Euro Surveill. (2020) 25:2000334. 10.2807/1560-7917.ES.2020.25.12.2000334 32234115 PMC7118342

[21] Thacker SB Stroup DF Sencer DJ. Epidemic assistance by the centers for disease control and prevention: role of the epidemic intelligence service, 1946–2005. Am J Epidemiol. (2011) 174 (11 Suppl):S4–15. 10.1093/aje/kwr307 22135393 PMC7109860

